# Frequent hypermethylation of orphan CpG islands with enhancer activity in cancer

**DOI:** 10.1186/s12920-016-0198-1

**Published:** 2016-08-12

**Authors:** Min Gyun Bae, Jeong Yeon Kim, Jung Kyoon Choi

**Affiliations:** Department of Bio and Brain Engineering, KAIST, Daejeon, 305-701 Republic of Korea

**Keywords:** CpG islands, Enhancers, DNA methylation, Cancer

## Abstract

**Background:**

CpG islands (CGIs) are interspersed DNA sequences that have unusually high CpG ratios and GC contents. CGIs are typically located in the promoter of protein-coding genes. They normally lack DNA methylation but become hypermethylated and induce repression of associated genes in cancer. However, the biological functions of non-promoter CGIs (orphan CGIs) largely remain unclear.

**Results:**

Here, we identify orphan CGIs that do not map to the promoter of any protein-coding or non-coding transcripts but possess chromatin and transcriptional marks that reflect enhancer activity (termed eCGIs). They exhibit three-dimensional chromatin looping toward multiple target genes with high affinity. Intriguingly, transcription regulators were frequently associated with such CGI-containing enhancers. Remarkably, our analyses in cell lines and clinical tissues showed that eCGIs have more dynamic DNA methylation changes in cancer relative to promoter CGIs. The observed eCGI hypermethylation was accompanied by a loss of enhancer marks and transcriptional inactivation of the target genes.

**Conclusion:**

Our results suggest that eCGIs may constitute a distinct class of enhancers and perform a more instrumental role in tumorigenesis than typical CGIs in gene promoters.

**Electronic supplementary material:**

The online version of this article (doi:10.1186/s12920-016-0198-1) contains supplementary material, which is available to authorized users.

## Background

CpG dinucleotides are frequently methylated in vertebrate genomes. Although a significant portion of the genome is methylated at CpG sites, CGIs are usually unmethylated and remain transcriptionally active with active histone marks such as H3K4me3 as a result of the action of CxxC finger protein 1 (CFP1) [1–4]. Half of these CGIs are located in gene promoters and play an important role in development and cancer. For example, important developmental genes have a promoter that often coincides with a CGI and contains a bivalent domain consisting of both active (H3K4me3) and repressive (H3K27me3) histone marks [5]. Those genes that have the bivalent promoter are marginally expressed in embryonic stem cells but increase in expression level via removal of the H3K27me3 mark during cell differentiation. Furthermore, the hypermethylation of promoter CGIs has been identified as one of the driving factors in cancer development because it represses the expression of tumor suppressor genes [6]. This phenomenon was first reported in the promoter of tumor suppressor genes in colorectal cancer and has been confirmed in many cancer types. In addition to promoter CGI hypermethylation, whole genome bisulfite sequencing has recently revealed partially methylated domains and large hypomethylated domains in cancer [7].

CGIs remote from annotated promoters, located in intergenic or intragenic regions, exhibit variable tissue-specific methylation patterns [8, 9]. These non-promoter CGIs are named orphan CGIs, and account for about half of all CGIs in the human genome [3]. Although these orphan CGIs are distal to annotated promoters, some features are shared with promoter CGIs: marking of H3K4me3, binding of Pol2, and production of transcripts, as indicated by a Cap Analysis of Gene Expression (CAGE) [3]. Recent studies suggest that these orphan CGIs may function as miRNA promoters [10], and therefore the presence of an orphan CGI is an important indicator of the activity of miRNA promoters [11]. Meanwhile, intragenic CGIs are known to act as an alternative promoter of the genes they reside in [8].

Although these recent studies propose that orphan CGIs may function as promoters, here we show that not all orphan CGIs produce transcripts, as judged by transcription start sites indicated by CAGE and RNA-seq. To understand the biological features and functions of the orphan CGIs that do not produce any noncoding transcripts, we perform an integrative analysis that entails a large amount of publicly available genomic, transcriptomic, and epigenomic data based on K562, Mcf7, and Hmec cell lines.

## Methods

### ENCODE data processing

Various histone modification, transcriptome, chromatin interactome, and DNA methylation data were downloaded from the ENCODE data portal (https://www.encodeproject.org). We downloaded bam files for various histone modifications including H3K4me1, H3K4me2, H3K4me3, H3K27ac, H3K27me3, H3K9me1, H3K9me3, H3K9ac, H3K79me2, H3K36me3, and H4K20me1. DNase I hypersensitivity site (DHS) data and transcription factor binding data for P300, Pol2, CTCF, RAD21, SMC3, YY1, and ZNF143 [12] were obtained as well. Peak finding for histone modifications and DHSs was performed using the HOMER package with -size 1000 and—minDist 2500 options.

CAGE and RNA-seq data were used to identify functional transcripts. We used the transcription start sites defined by CAGE. RNA-seq fastq files were aligned by using Tophat and de-novo transcripts were predicted by running StringTie [13] with its default options. Gene expression in each cell line was then determined based on Reads Per Kilobase per Million (RPKM).

Chromatin interactome in K562 and Mcf7 cell lines were analyzed based ona Chromatin Interaction Analysis by paired-end tag (ChIA-pet) sequencing data for RNA polymerase II (Pol2). In order to use significant interactions only, tag counts greater or equal to 3 were taken.

### Classification of CGIs

To classify CGIs based on gene annotation, we selected genes whose refGene ID starts with “NM”. The CGIs that are located within 1 kb of the transcription start site of the relevant genes were labeled as promoter CGIs (pCGIs), and the rest as orphan CGIs. The orphan CGIs that overlap with both H3K27ac and DHS peaks in a given cell type were then determined as active orphan CGIs. By checking whether the active orphan CGIs overlap with the transcription start sites defined in the CAGE data and the promoter of de-novo transcripts constructed from the RNA-seq data using StringTie, we defined eCGIs as not producing any protein-coding or non-coding transcripts, and npCGIs (noncoding promoter CGIs) as producing non-coding transcripts.

Typical enhancers were defined as H3K27ac-harboring DHS peaks that do not overlap with the transcription start site of de-novo transcripts detected from the CAGE and RNA-seq data. We also excluded the H3K27ac-DHS peaks intersecting with any CGIs. Using this method, we found 9282, 18,528, 20,332 typical enhancers in K562, Mcf7, and Hmec, respectively.

### Target gene analysis

To check the function of the target genes of the eCGIs, we used the ‘functional annotation clustering’ of the Database for Annotation, Visualization and Integrated Discovery (DAVID) with the default options. The annotation clusters with the highest enrichment scores seems to be related to transcription.

To confirm that the eCGIs target transcription regulators, we used the list of 1469 sequence-specific transcription factors, 117 chromatin regulators, and 296 transcription-related factors as defined in the AnimalTFDB data [14] for *Homo sapiens*. We obtained the number of transcription regulators that are linked via chromatin interaction to the eCGIs or typical enhancers. To estimate the statistical significance of the overlapping, we selected the same number of random DNA segments as the eCGIs and typical enhancers in each cell type, and compared their overlapping frequencies with that of the real eCGIs and typical enhancers.

### DNA methylation analysis

To investigate DNA methylation changes in association with the eCGIs, we used breast related normal and cancer pair (Hmec and Mcf7) and The Cancer Gene Atlas (TCGA; http://cancergenome.nih.gov) data. Heatmaps were generated using differentially methylated CpGs of the Hmec eCGIs in both cell lines and clinical data. A threshold of differentially methylated CpGs was determined as |differential methylation of CpGs| > 0.5 in the cell line data and |differential methylation of CpGs| > 0.1 in the clinical data. To determine the target genes of the Hmec eCGIs, we used the genes in the nearest proximity to the eCGIs due to lack of Hmec ChIA-pet data. Enrichment test of tumor suppressor genes and oncogenes were performed using Fisher-exact test. Tumor suppressor genes and oncogenes used in this study were generated by the TUSON algorithm [15]. We extracted tumor suppressor genes (484) and oncogenes (494) with low *p*-value (<0.1).

## Results

### Classification of CGIs

**Fig. 1:**
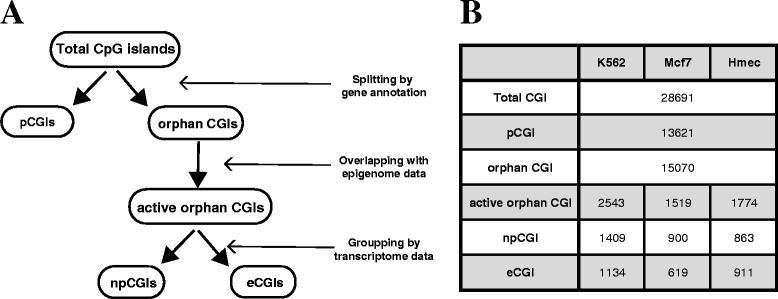
Classification of CGIs. **a** Diagram of CGI classification. pCGI refers to promoter CGIs, npCGI refers to noncoding promoter CGIs, and eCGI refers to enhancer CGIs. **b** The number of CGIs in each cell line

We classified CGIs based on the pipeline that interrogates gene annotation, epigenome data, and transcriptome data (Fig. 1a). To identify cell-type-specific functional CGIs, DHS and H3K27ac patterns in K562, Mcf7, and Hmec cell lines were used. DHSs indicate open regulatory sites and H3K27ac is an active histone marker that is usually found on regulatory sites such as promoters and enhancers. According to these criteria, an average of 5.3 ~ 8.9 % of total CGIs were identified as active orphan CGIs in each cell line. Because orphan CGIs are associated with the transcription of noncoding RNA, we checked whether they actually make transcripts by analyzing CAGE and RNA-seq profiles. The hidden Markov model was applied to the CAGE data to identify regions that can function as a transcription start site. In addition, RNA-seq was used to identify de-novo transcripts. While 863 ~ 1409 CGIs were associated with non-coding transcripts and labeled as npCGIs, 619 ~ 1134 CGIs were classified as eCGIs because they did not map to any transcription start sites despite having active enhancer marks (Fig. 1b).

This new type of CGIs does not show genomic characteristics shared with the pCGIs. First, the eCGIs are shorter in length than the pCGIs (Additional file 1: Figure S1A). Longer pCGIs may have been favored during evolution because they are well-suited for multiple transcription factors to bind and are related with promoter directionality [16, 17]. Although well-established CGI criteria based on the CpG ratio and GC percent were used, the sequence contents of the pCGIs and eCGIs appear to be different. Compared to the pCGIs, the eCGIs have a lower CpG ratio and CpG percent, and the GC percent differs statistically significantly (Additional file 1: Figure S1B). In other words, the eCGIs have a higher frequency of C and G, but the ratio of CpG sites, which can be methylated, is lower in the eCGIs than in the pCGIs. Although specific mechanisms leading to this discrepancy are currently unknown, it is evident that the eCGIs have distinct genomic features as compared with the pCGIs.

### Enhancer signatures of eCGIs

**Fig. 2:**
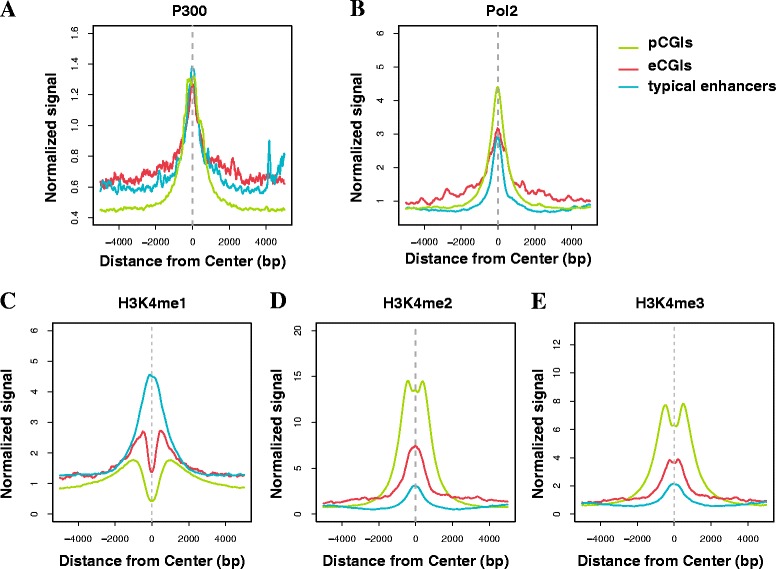
Epigenetic marks reveal that the eCGIs have enhancer signatures. Distribution of epigenetic marks spanning 5 kb from the center of the eCGIs, typical enhancers, and pCGIs, respectively. P300 and Pol2 are closely related to enhancer activity **a**, **b** and H3K4 methylations are known as enhancer **c**, **d** or promoter **e** markers

As described above, we predicted that the identified eCGIs would function as enhancers because they were enriched for H3K27ac while not mapping to any transcription start sites. In order to corroborate our prediction, the binding level of P300, which is a histone acetyl transferase known as an enhancer marker, was measured. Our results verified that the P300 binding level was similar between the eCGIs and typical enhancers (Fig. 2a).

Next, we examined the binding level of Pol2. Studies indicate that Pol2 transcribes not only mRNA but also noncoding RNA, and that it binds at enhancer regions as well. While the strongest Pol2 binding was observed at the pCGIs, the Pol2 binding levels were similar in the eCGIs and typical enhancers (Fig. 2).

Additionally, we examined the distribution of various histone modifications. H3K4me1 and H3K4me3 are well-established enhancer and promoter markers, respectively [18]. We discovered that H3K4me1 was enriched in the typical enhancers while H3K4me3 in the pCGIs. In eCGIs, H3K4me1 was highly enriched, to a degree comparable with the typical enhancers (Fig. 2c). Although the middle point of the eCGIs showed a signature of nucleosome depletion, the H3K4me1 levels were higher at the boundary areas. The H3K4me2 and H3K4me3 distributions showed intermediate values between the pCGIs and typical enhancer (Fig. 2d, e). Previous studies suggest that CFP1 binds to sequences with high CpG contents and recruits SETD1, which causes trimethylation of H3K4 [4]. This might explain high H3K4me3 in eCGIs regardless of their promoter activity. Taken together, the results indicate that the eCGIs are similar to the typical enhancers in terms of chromatin signatures.

We also examined other histone modifications. H3K9ac, an active promoter marker, was high in the pCGIs. H3K79me2, an elongation marker that is strongly enriched in the first intron, was also high in the pCGIs. H3K9me1, H4K20me1, and H3K36me3 showed marginal enrichment in the typical enhancer. Because these three histone marks are related with transcription elongation, this may be a reflection of the typical enhancers residing in the genebody. Repressor markers such as H3K27me3 and H3K9me3 did not show any enrichment patterns (Additional file 1: Figure S2A).

For the eCGIs to have an enhancer function, they should interact with the transcription start site or the promoter of their target genes. Five proteins, CTCF, RAD21, YY1, ZAN143, and SMC3, are known to govern such chromatin interactions. All five proteins were enriched in the eCGIs; in particular, CTCF and SMC3 signals were much stronger in the eCGIs than in the pCGIs and the typical enhancers (Additional file 1: Figure S2B).

### Transcriptional activity of eCGIs

**Fig. 3:**
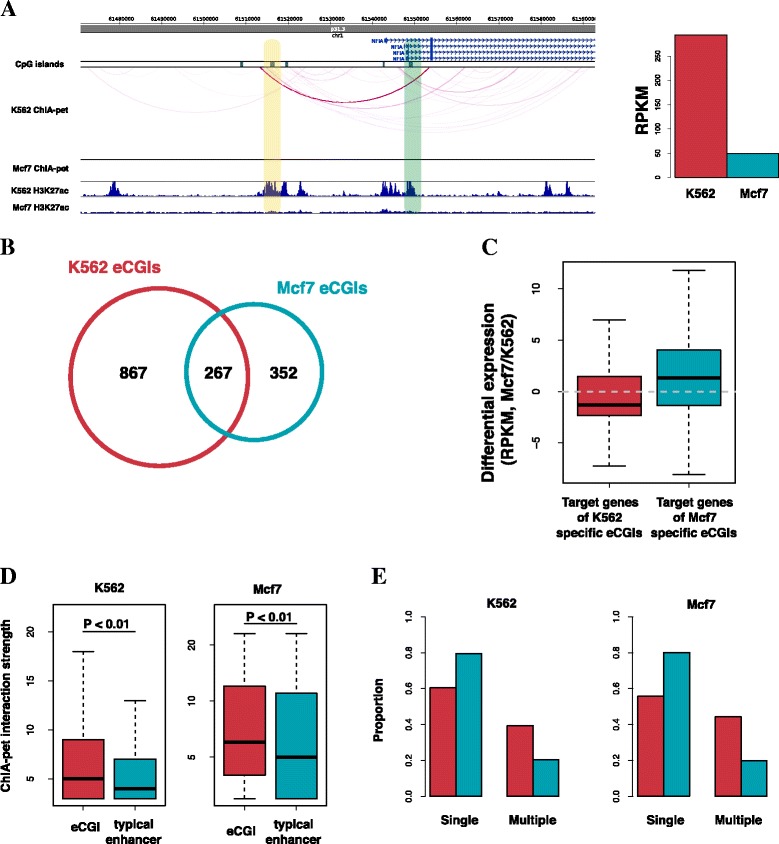
eCGIs are a distinct class of enhancers that regulate target gene expression. **a** The left panel is an example of a cell-type-specific eCGI target, demonstrated using the WashU epigenome browser. The yellow shaded region indicates K562-specific eCGIs and the green shaded region represents their target gene, NFIA. The right panel compares the gene expression level (RPKM) of NFIA in K562 and Mcf7 cell lines. **b** The diagram shows overlapping between eCGIs and npCGIs in K562 and Mcf7 cell lines. **c** Expression fold change of genes targeted by each cell-type-specific eCGI. **d** ChIA-pet signal distribution in K562 (left) and Mcf7 (right) cell lines. The *P*-value was calculated by the Wilcoxon signed-rank test. **e** Histogram showing the proportion of enhancers targeting one gene (single) and two or more genes (multiple)

We next sought to test whether the eCGIs regulate the expression of their target gene. To this end, we first identified the eCGIs that are active specifically in K562 and Mcf7: 867 (76.5 %) were specific to K562, 352 (56.9 %) were specific to Mcf7, and 267 were common (Fig. 3b, Additional file 1: Figure S3). We discovered that the cell-type-specific eCGIs were associated with differential up regulation of their target gene connected via chromatin interaction, as identified through ChIA-pet (Fig. 3c). For example, NFIA gene, one of the nuclear factor I family, is known as a transcription factor that plays an important function in the brain, and ureteral and renal development and hematopoiesis [19]. This gene has ChIA-pet chromatin interaction with an eCGI in K562 but no interaction in Mcf7. The expression level of this gene was 5.88-fold higher in K562 than in Mcf7 (Fig. 3a). These results suggest that the eCGIs we identified may function as enhancers that induce activation of their target gene.

### ECGIs as a distinct class of enhancers

**Fig. 4:**
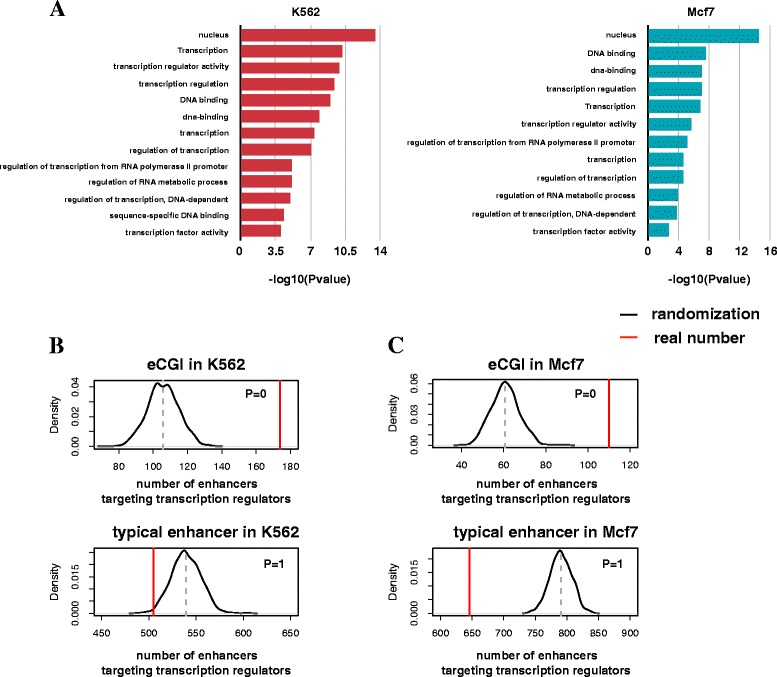
eCGIs primarily target transcription regulators. **a** The gene clusters with the highest enrichment score detected by the DAVID’s functional annotation clustering in K562 (left) and Mcf7 (right). P values were transformed by –log10. All clustering lists are provided in Additional file 1: Table S2. **b**, **c** The distribution of the number of transcription regulators associated with random DNA fragments (black line). The permutation was repeated 1000 times. Red lines indicate the number of transcription regulators associated with the eCGIs (upper) or typical enhancers (lower) in K562 (B) and in Mcf7 (C)

We substantiated that the eCGIs have enhancer activity by examining 1) P300 binding, 2) histone modification patterns, 3) chromatin interaction with promoters, and 4) transcriptional activity. We next concentrated on differences between the eCGIs and typical enhancers. We first examined the features of chromatin interaction involving either eCGI or a typical enhancer. The average ChIA-pet tag count for each pair of the eCGIs and their connected promoters was significantly higher than that for each pair of typical enhancers and their promoters (Fig. 3d). The eCGIs were more active than the typical enhancers not only in terms of interaction strength but also in terms of the number of the interactions (Fig. 3e). Thus, the eCGIs may play a more pivotal role in regulating gene expression than the typical enhancers. We examined Gene Ontology to characterize the target genes of the eCGIs. The strongest functional enrichment was observed for transcription in both K562 and Mcf7 (Fig. 4a). For example, the NFIA gene described above is known to play diverse roles as a transcription factor across many cell types (Fig. 3a). For a statistical test, we performed 1000 permutations to obtain the expected number of chromatin interactions between transcription factors and the eCGIs or typical enhancers. The same number of random DNA segments as the eCGIs or typical enhancers in K562 and Mcf7 were generated. We then examined the number of links with transcription regulators such as sequence-specific transcription factors, chromatin regulators, and transcription related factors as defined in the AnimalTFDB [14]. As a result, in both cell lines, the eCGIs were more frequently associated with the transcription regulators than expected by chance (Fig. 4b, c). In contrast, the typical enhancers were less frequently associated than expected by chance (Fig. 4b, c).

Previous studies have identified super enhancers, also known as stretch enhancers, which are large clusters of transcriptional enhancers that drive expression of genes that define cell identity [20, 21]. We checked whether the eCGIs we identified here are coincident with the super enhancers. In terms of physical overlapping in K562, only a small fraction (9.7 %) of the eCGIs was previously identified as super enhancers (Additional file 1: Figure S4A). H3K27ac is the major histone mark that is used to identify super enhancers. The H3K27ac levels were much lower in the eCGIs than in the super enhancers (Additional file 1: Figure S4B). Taken altogether, the results suggest that the eCGIs constitute a new type of enhancers that are different from typical enhancers or super enhancers.

### Dynamic tumorigenic changes of DNA methylation at eCGIs

**Fig. 5:**
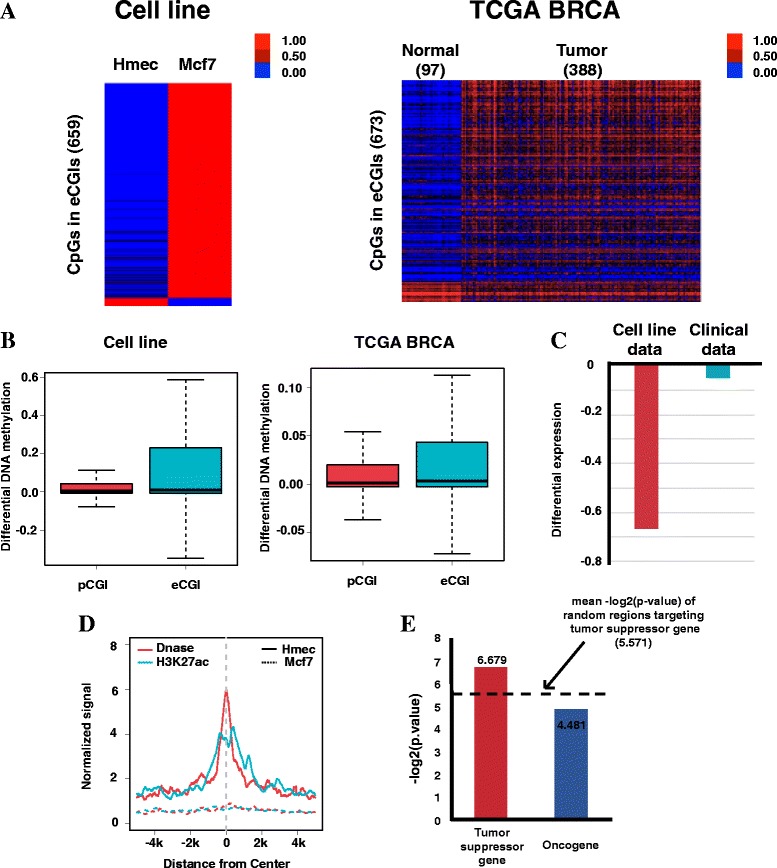
Dynamic tumorigenic changes of DNA methylation in the eCGIs. **a** Heatmap showing aberrant DNA methylation of the eCGIs in Mcf7 cell line (left) and clinical cancer data (right). **b** Boxplots showing that the eCGIs are more hypermethylated than the pCGIs in both cell lines (left) and clinical data (right). **c** Down-regulation of the target genes of the hypermethylated eCGIs in Mcf7 cell line and clinical cancer data. **d** Global loss of DHS and H3K27ac signals at hypermethylated eCGIs in Mcf7. **e** Fisher-exact test for enrichment of target genes of hypermethylated eCGIs on tumor suppressor genes

In cancer, tumor suppressor genes are inactivated by pCGI hypermethylation. To study whether DNA methylation at eCGIs play a role in cancer, we analyzed the ENCODE methylation data of Hmec and Mcf7 cell lines and the TCGA breast-normal cancer data. The overall pattern of the heatmaps showed that the methylation increases at the eCGIs in cancer cells when compared to normal cells and the differential DNA methylation levels across the cell lines and clinical data were significantly higher in the eCGIs than in the pCGIs (Fig. 5a, b). This may indicate that particular eCGIs are hypermethylated to a higher degree than an average pCGI in a cancer-specific manner. This aberrant DNA methylation in the eCGIs may play a more critical role in tumorigenesis than that in the pCGIs.

To test whether the oncogenic DNA methylation changes in the eCGIs affect regulatory activities and ultimately gene expression levels, we selected eCGIs with a > 0.5 DNA methylation increase in Mcf7 compared to Hmec, and those with a > 0.1 DNA methylation increase in clinical cancer samples compared to normal samples. The target gene expression level of hypermethylated eCGIs was lower in Mcf7 and clinical cancer data, suggesting transcriptional silencing effects of eCGI hypermethylation in cancer (Fig. 5c). To study the mechanism of transcriptional silencing effects of eCGI hypermethylation, we used DHS and H3K27ac signal in Hmec and Mcf7. The hypemethylation of these eCGIs was accompanied by a significant reduction in the DHS and H3K27ac signals (Fig. 5d), suggesting that some eCGIs function as enhancers in normal cells but lose their enhancer function due to DNA hypermethylation in cancer. To test that genes silenced by eCGI hypermethylation are tumor suppressor genes, we performed Fisher-exact test using 484 predicted tumor suppressor genes and 494 predicted oncogenes (Fig. 5e). The enrichment of targeted tumor suppressor genes by hypermethylated eCGIs is higher than the enrichment of targeting tumor suppressor genes by random regions and targeting oncogenes by eCGIs. This indicates that eCGI hypermethylation may inactivate tumor suppressor genes by removing enhancer activity.

## Discussion

An enhancer is a distal regulatory region that activates the expression of remote genes. A global epigenome study reveals that enhancers usually are bound by P300 proteins, and possess H3K4me1 and H3K27ac marks but not H3K4me3, which is known as a promoter marker. However, recent studies show that some active enhancers possess H3K4me3. Also, eRNAs that are made by polymerase II bound at enhancer regions stabilize enhancer-promoter looping. Based on the fact that eRNAs are transcribed bidirectionally, active enhancers were detected using the bidirectional CAGE distribution through FANTOM5 [22].

CpG islands are DNA sequences having high CpG ratios and GC contents. About half of CpG islands are located around transcription start site of protein coding genes. Promoters with CpG islands have higher expression than non-CpG island promoters and housekeeping genes usually have promoter CpG islands. These promoter CpG islands are usually hypermethylated during tumorigenesis and inactivate tumor suppressor genes. However, the other half of CpG islands that are not located at promoter regions have not been studied extensively. Some studies reported that some orphan CpG islands are promoters of noncoding RNAs, such as microRNA and lncRNA, through CAGE and RNA-seq analyses. Further study is still required.

Here we identified that some of these orphan CGIs possess the characteristics of enhancers, including H3K4me1, H3K27ac, P300 binding, three-dimensional interaction, and transcriptional activity. These eCGIs differ from the typical promoter CGIs not only in terms of genomic features, such as CGI size and sequence contents, but also in terms of epigenomic features such as the intensity patterns of particular histone modifications. The enhancers harboring a CGI were also different from typical enhancers. They are capable of interacting with multiple target genes with a higher chromatin interaction affinity. Intriguingly, most of these targeted genes are transcription regulators. Thus, the eCGIs appear to play a more important role in the regulatory network. Importantly, the eCGIs tend to be hypermethylated during cancer development in both cell lines and clinical breast cancer samples. Although this is a well established feature of the typical promoter CGIs, the degree of DNA methylation changes is greater for the eCGIs than the typical CGIs. Because of hypermethylation in eCGIs, enhancer signatures disappear and down regulate target genes.

## Conclusion

We identified eCGIs using various epigenomic and transcriptomic features based on H3K27ac sequencing, RNA-seq, and CAGE data. This method may produce false positives even though eCGIs that we found have enhancer activity. To overcome this problem, STARR-seq [23], which can detect enhancers quantitatively, may be useful to find eCGIs. We also found that eCGIs are frequently hypermethylated in both cell lines and clinical tissues. This suggests that orphan CGIs with enhancer activity in a given cell type should be considered a novel biomarker in cancer diagnosis and treatment.

## Additional files

Additional file 1: Figure S1. Differences in genetic characteristics such as CpG island size, CpG ratio, CpG percent, and GC percent between pCGIs and eCGIs. **Figure S2.** Comparison of histone modifications and chromatin interaction factors between pCGIs, eCGIs and typical enhancers. **Figure S3.** Overlapping between eCGIs and npCGIs in K562 and Mcf7 cell lines. **Figure S4.** Comparison between eCGIs and super enhancers (PDF 519 kb)

## References

[1] Guenther MG, Levine SS, Boyer L a, Jaenisch R, Young R a (2007). A chromatin landmark and transcription initiation at most promoters in human cells. Cell.

[2] Mikkelsen TS, Ku M, Jaffe DB, Issac B, Lieberman E, Giannoukos G (2007). Genome-wide maps of chromatin state in pluripotent and lineage-committed cells. Nature.

[3] Illingworth RS, Gruenewald-Schneider U, Webb S, Kerr ARW, James KD, Turner DJ (2010). Orphan CpG islands identify numerous conserved promoters in the mammalian genome. PLoS Genet.

[4] Thomson JP, Skene PJ, Selfridge J, Clouaire T, Guy J, Webb S (2010). CpG islands influence chromatin structure via the CpG-binding protein Cfp1. Nature.

[5] Bernstein BE, Mikkelsen TS, Xie X, Kamal M, Huebert DJ, Cuff J (2006). A bivalent chromatin structure marks Key developmental genes in embryonic stem cells. Cell.

[6] Esteller M (2002). CpG island hypermethylation and tumor suppressor genes: a booming present, a brighter future. Oncogene.

[7] Berman BP, Weisenberger DJ, Aman JF, Hinoue T, Ramjan Z, Liu Y (2011). Regions of focal DNA hypermethylation and long-range hypomethylation in colorectal cancer coincide with nuclear lamina—associated domains. Nat Genet.

[8] Maunakea AK, Nagarajan RP, Bilenky M, Ballinger TJ, D’Souza C, Fouse SD (2010). Conserved role of intragenic DNA methylation in regulating alternative promoters. Nature.

[9] Illingworth R, Kerr A, Desousa D, Jørgensen H, Ellis P, Stalker J (2008). A novel CpG island set identifies tissue-specific methylation at developmental gene loci. PLoS Biol.

[10] Ozsolak F, Poling LL, Wang Z, Liu H, Liu XS, Roeder RG (2008). Chromatin structure analyses identify miRNA promoters chromatin structure analyses identify miRNA promoters. Genes Dev.

[11] Marsico A, Huska MR, Lasserre J, Hu H, Vucicevic D, Musahl A (2013). PROmiRNA: a new miRNA promoter recognition method uncovers the complex regulation of intronic miRNAs. Genome Biol.

[12] Rao SSP, Huntley MH, Durand NC, Stamenova EK: A 3D Map of the Human Genome at Kilobase Resolution Reveals Principles of Chromatin Looping. *Cell* 2014;159:1–16. 10.1016/j.cell.2014.11.021 PMC5635824 25497547

[13] Pertea M, Pertea GM, Antonescu CM, Chang T-C, Mendell JT, Salzberg SL: StringTie enables improved reconstruction of a transcriptome from RNA-seq reads. *Nat Biotechnol* 2015;33:290-5. 10.1038/nbt.3122 PMC4643835 25690850

[14] Zhang HM, Chen H, Liu W, Liu H, Gong J, Wang H (2012). AnimalTFDB: a comprehensive animal transcription factor database. Nucleic Acids Res.

[15] Chen K, Chen Z, Wu D, Zhang L, Lin X, Su J (2015). Broad H3K4me3 is associated with increased transcription elongation and enhancer activity at tumor-suppressor genes. Nat Genet.

[16] Elango N, Yi SV (2011). Functional relevance of CpG island length for regulation of gene expression. Genetics.

[17] Almada AE, Wu X, Kriz AJ, Burge CB, Sharp P a (2013). Promoter directionality is controlled by U1 snRNP and polyadenylation signals. Nature.

[18] Heintzman ND, Hon GC, Hawkins RD, Kheradpour P, Stark A (2009). Histone modifications at human enhancers reflect global cell-type-specific gene expression. Nature.

[19] Bernard F, Gelsi-Boyer V, Murati a, Giraudier S, Trouplin V, Adélaïde J (2009). Alterations of NFIA in chronic malignant myeloid diseases. Leuk Off J Leuk Soc Am Leuk Res Fund UK.

[20] Whyte W, Orlando D a, Hnisz D, Abraham BJ, Lin CY, Kagey MH (2013). Master transcription factors and mediator establish super-enhancers at key cell identity genes. Cell.

[21] Hnisz D, Abraham BJ, Lee TI, Lau A, Saint-André V, Sigova A a (2013). Super-enhancers in the control of cell identity and disease. Cell.

[22] Andersson R, Gebhard C, Miguel-Escalada I, Hoof I, Bornholdt J, Boyd M (2014). An atlas of active enhancers across human cell types and tissues. Nature.

[23] Arnold CD, Gerlach D, Stelzer C, Boryn LM, Rath M, Stark A (2013). Genome-wide quantitative enhancer activity maps identified by STARR-seq. Science.

