# Supplementary material for: Frequent hypermethylation of orphan CpG islands with enhancer activity in cancer
Source: BMC Med Genomics. 2016 Aug 12;9(Suppl 1):38. doi: 10.1186/s12920-016-0198-1 (PMC4989897; doi:10.1186/s12920-016-0198-1)

Supplementary figure 1

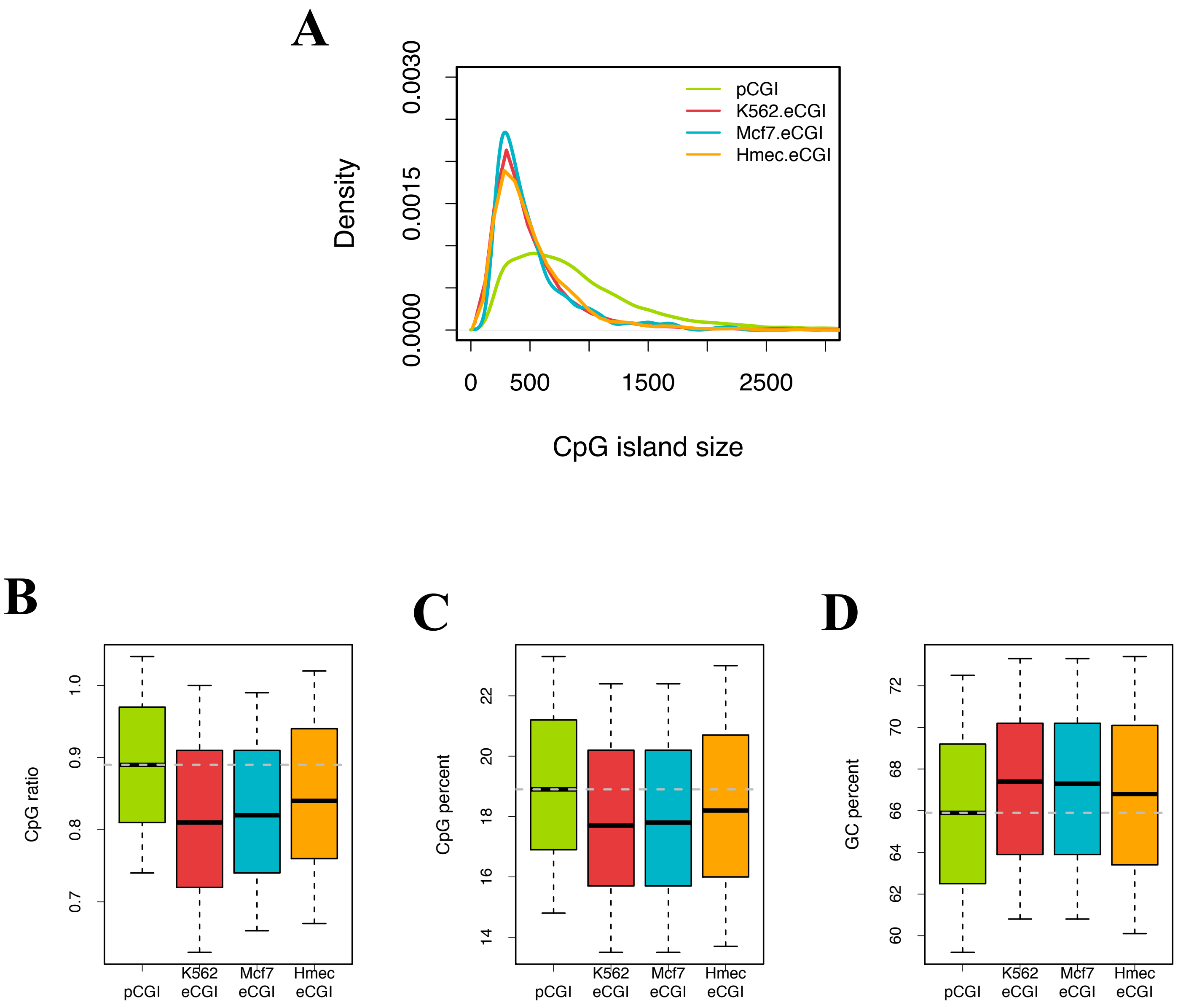

Supplementary figure 2

A

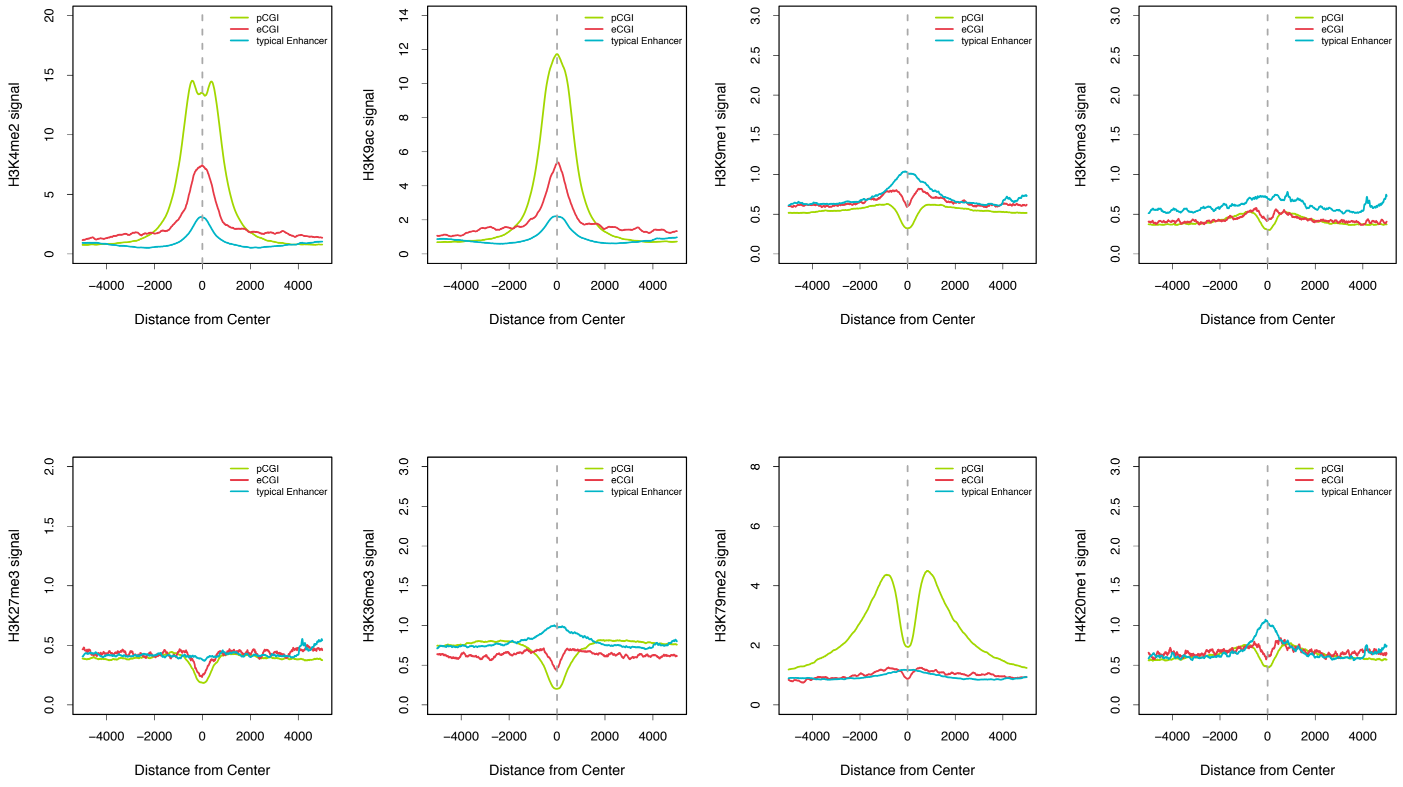

B

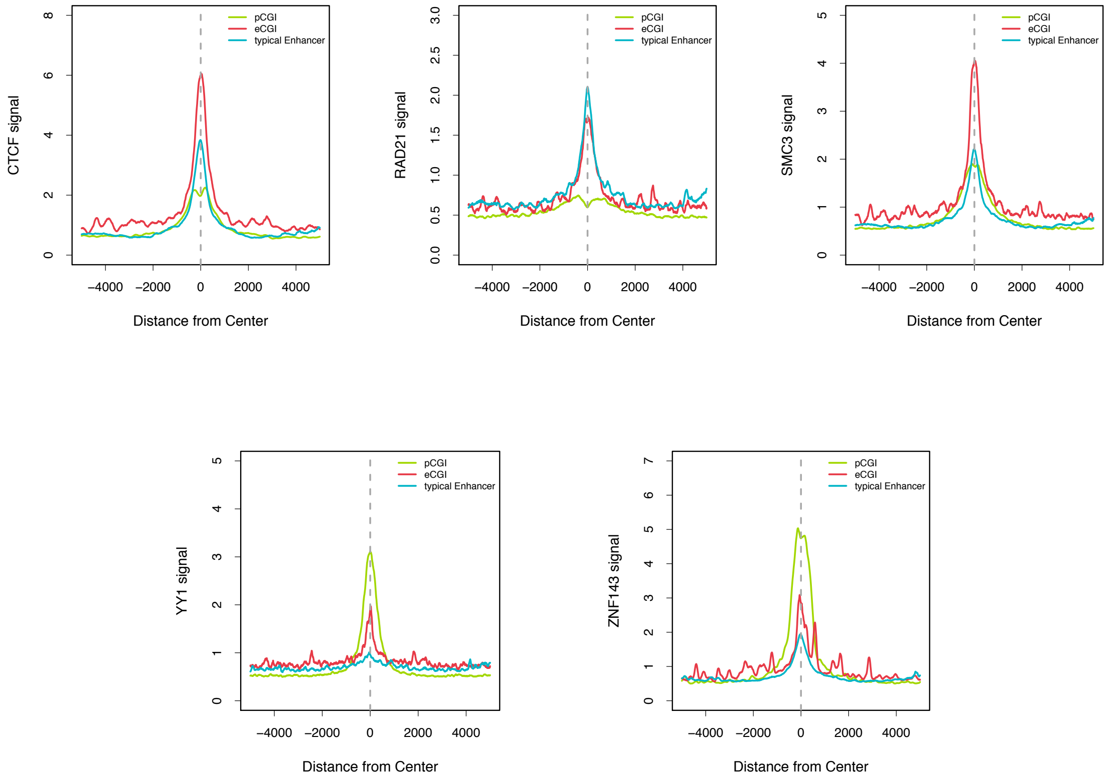

Supplementary figure 3

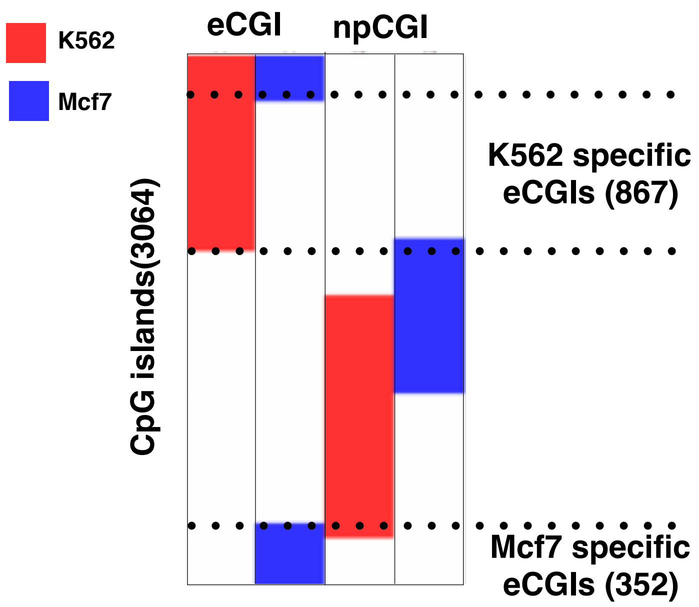

Supplementary figure 4

A

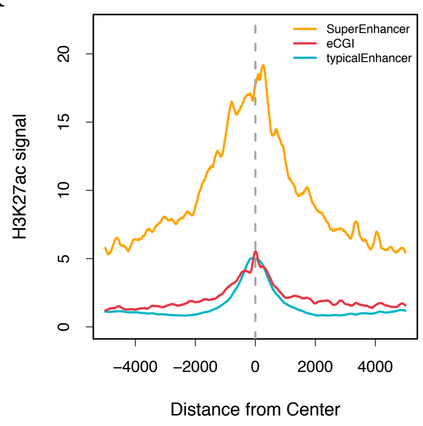

B

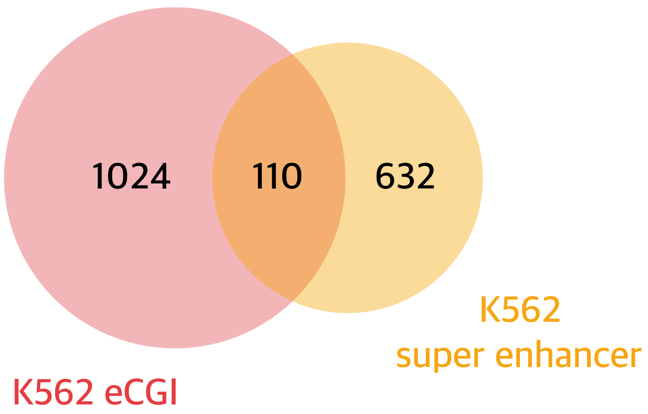

Supplement: Additional file 1: Figure S1. — Differences in genetic characteristics such as CpG island size, CpG ratio, CpG percent, and GC percent between pCGIs and eCGIs. Figure S2. Comparison of histone modifications and chromatin interaction factors between pCGIs, eCGIs and typical enhancers. Figure S3. Overlapping between eCGIs and npCGIs in K562 and Mcf7 cell lines. Figure S4. Comparison between eCGIs and super enhancers (PDF 519 kb) [file 12920_2016_198_MOESM1_ESM.pdf]
